# Supplementary material for: Role of law enforcement agencies in suicide prevention: A scoping review
Source: Glob Ment Health (Camb). 2026 Mar 30;13:e71. doi: 10.1017/gmh.2026.10186 (PMC13112288; doi:10.1017/gmh.2026.10186)
Supplement: Jain et al. supplementary material 2 — Jain et al. supplementary material [file S2054425126101861sup002.docx]

**Search Strategy for Each Database**

**PubMed / MEDLINE and Embase**

**Via EMBASE (OVID)**

**CONCEPT 1- Suicide Prevention Processes**

("suicide*" OR "self-harm*" OR "suicidal" OR "suicidality" OR "parasuicide" OR "self-directed violence" OR "parasuicidal" OR "self-injurious behaviour" OR "self-destructive behaviour" OR "self-killing" OR "self-injur*" OR "self-mutilat*" OR "self-immolat*" OR "self-poison*" OR "self-drowning" OR "self-hang*" OR "deliberate overdose" OR "deliberate self-harm" OR "suicide attempt*" OR "intentional self-harm" OR "suicide prevention" OR "suicide ideation")

NOT

("assisted dying" OR "euthanasia" OR "unintentional self-injur*" OR "unintentional self-harm" OR "physician assisted death" OR "non-deliberate self-injur*" OR "non-deliberate self-harm")

AND

("intervention*" OR "prevent*" OR "crisis intervention" OR "emergency response" OR "early intervention" OR "preventative measure*" OR "risk reduction" OR "harm reduction" OR "crisis intervention" OR "primary prevention" OR "secondary prevention" OR "tertiary prevention" OR "community intervention" OR "preventive strategies")

AND

("training" OR "capacity building" OR "skill development" OR "professional development" OR "workforce development" OR "education" OR "knowledge transfer" OR "competency building" OR "capacity building" OR "education" OR "awareness program*" OR "training programs" OR "training initiatives" OR "educational intervention")

AND

(polic* OR guideline* OR strateg* OR "best practice*" OR "evidence-based practice*" OR protocol* OR "standard operating procedure*" OR framework* OR "action plan*" OR "implementation plan*" OR "practice guideline*" OR "health polic*" OR "policy recommendations" OR "policy development" OR "policy implementation")

**CONCEPT 2 – Law Enforcement**

("law enforcement system*" OR "law enforcement agenc*" OR "law enforcement officer*" OR "law enforcement agent*" OR police* OR policing OR "criminal justice" OR "police force*" OR "police officer*" OR "law enforcement training" OR "law enforcement intervention" OR "law enforcement practices" OR "law enforcement policies" OR "law enforcement strategies" OR "criminal justice system" OR "criminal justice intervention")

**PsycINFO**

**CONCEPT 1- Suicide Prevention Processes**

1.PubMed/ PsycInfo Key words – “suicide*”  OR “self-harm*”  OR “suicidal”  OR “suicidality”  OR “parasuicide”  OR “self-directed violence”  OR parasuicidal OR “self-injurious behaviour” OR “self-destructive behaviour” OR  "self-killing"  OR self-injur*  OR self-mutilat*  OR self-immolat*  OR self-poison*  OR self-drowning  OR self-hang*  OR "deliberate overdose"  OR “deliberate self-harm”  NOT (“non-suicidal self injur*” 'euthanasia'/exp OR "assisted dying":ti,ab,kw OR "unintentional self-injur*":ti,ab,kw OR "unintentional self-harm":ti,ab,kw OR "physician assisted death":ti,ab,kw OR "non-deliberate self-injur*":ti,ab,kw OR "non-deliberate self-harm":ti,ab,kw)

AND

((DE "Crisis Intervention" OR DE "Prevention") OR (intervention* OR prevent* OR "crisis intervention" OR "emergency response" OR "early intervention" OR "preventive measure*" OR "preventative measure*" OR "risk reduction" OR "harm reduction")) AND ((DE "Training") OR (training OR "capacity building" OR "skill development" OR "professional development" OR "workforce development" OR education OR "knowledge transfer" OR "competency building")) AND ((DE "Professional Standards") OR (polic* OR guideline* OR strateg* OR "best practice*" OR "evidence-based practice*" OR protocol* OR "standard operating procedure*" OR framework* OR "action plan*" OR "implementation plan*"))

Mesh terms- "Suicide"[Mesh] OR "Suicide, completed"[Mesh] OR "Suicide, Attempted"[Mesh] OR "Self-Injurious Behavior"[Mesh]

**CONCEPT 2 – Law Enforcement Personnel**

1. PubMed/PsycInfo Key words- "law enforcement officer*"  OR "law enforcement agent*"  OR police*  OR policing  OR “criminal justice”  OR “police force*”  OR “police officer*” [tiab]

Mesh terms- "Law Enforcement Officers"[Mesh] OR "Enforcement Officer, Law"[Mesh] OR "Enforcement Officers, Law" [Mesh] OR "Law Enforcement Officer"[Mesh] OR "Officer, Law Enforcement"[Mesh] OR "Officers, Law Enforcement"[Mesh] OR "Police Force"[Mesh] OR "Police Forces"[Mesh] OR "Police Officers"[Mesh] OR “Officers, Police”[Mesh]OR "Officer, Police"[Mesh]

**Google Scholar**

**CONCEPT 1- Suicide Prevention Processes**

“suicide prevention” - euthanasia

**CONCEPT 2 – Law Enforcement Personnel**

 police

**CINAHL**

**CONCEPT 1- Suicide prevention processes**

(MH "Suicide+") OR (MH "Suicidal Ideation") OR (MH "Suicide, Attempted") OR TI (suicid* OR "self-harm" OR "self harm" OR "self-injury" OR "self injury" OR "self-directed violence" OR "suicidal ideation" OR "suicidal behavior" OR "suicidal behaviour" OR "suicide attempt*" OR "attempted suicide" OR "self-poisoning" OR "self poisoning") NOT ("assisted dying" OR euthanasia OR "unintentional self-injur*" OR "unintentional self-harm" OR "physician assisted death" OR "non-deliberate self-injur*" OR "non-deliberate self-harm")

AND

((MH "Crisis Intervention") OR (MH "Preventive Health Care") OR intervention* OR prevent* OR "crisis intervention" OR "emergency response" OR "early intervention" OR "preventive measure*" OR "preventative measure*" OR "risk reduction" OR "harm reduction") AND ((MH "Education") OR training OR "capacity building" OR "skill development" OR "professional development" OR "workforce development" OR education OR "knowledge transfer" OR "competency building") AND ((MH "Practice Guidelines") OR polic* OR guideline* OR strateg* OR "best practice*" OR "evidence-based practice*" OR protocol* OR "standard operating procedure*" OR framework* OR "action plan*" OR "implementation plan*")

**CONCEPT 2 – Law Enforcement Personnel**

CINAHL: ((MH "Police+") OR (MH "Law Enforcement+") OR "peace officer*" OR constable* OR "public safety officer*" OR "security force*")

**Web of Science**

**CONCEPT 1- Suicide prevention processes**

TS=( (suicid* OR "self-harm" OR "self harm" OR "self-injury" OR "self injury" OR "self-directed violence" OR "suicidal ideation" OR "suicidal behavior" OR "suicidal behaviour" OR "suicide attempt*" OR "attempted suicide" OR "self-poisoning" OR "self poisoning") NOT TS=("assisted dying" OR euthanasia OR "unintentional self-injur*" OR "unintentional self-harm" OR "physician assisted death" OR "non-deliberate self-injur*" OR "non-deliberate self-harm")

AND

TS=( (intervention* OR prevent* OR "crisis intervention" OR "emergency response" OR "early intervention" OR "preventive measure*" OR "preventative measure*" OR "risk reduction" OR "harm reduction") AND (training OR "capacity building" OR "skill development" OR "professional development" OR "workforce development" OR education OR "knowledge transfer" OR "competency building") AND (polic* OR guideline* OR strateg* OR "best practice*" OR "evidence-based practice*" OR protocol* OR "standard operating procedure*" OR framework* OR "action plan*" OR "implementation plan*") )

**CONCEPT 2 – Law Enforcement Personnel**

TS=((police* OR "law enforcement" OR "peace officer*" OR constable* OR "public safety officer*" OR "security force*")

**Scopus**

**CONCEPT 1- Suicide prevention processes**

TITLE-ABS-KEY {suicide prevention}

AND

policies OR intervention OR guidelines OR training

**CONCEPT 2 – Law Enforcement Personnel**

TITLE-ABS-KEY {law enforcement} OR police OR {law enforcement system}

**Google**

**CONCEPT 1- Suicide prevention processes**

 "suicide prevention" -euthanasia

**CONCEPT 2 – Law Enforcement Personnel**

police

 
 
 
